# Influence of Different Tetracycline Antimicrobial Therapy of Mycoplasma (*Mycoplasma synoviae*) in Laying Hens Compared to Tea Tree Essential Oil on Table Egg Quality and Antibiotic Residues

**DOI:** 10.3390/foods9050612

**Published:** 2020-05-11

**Authors:** Nikola Puvača, Erinda Lika, Vincenzo Tufarelli, Vojislava Bursić, Dragana Ljubojević Pelić, Nedeljka Nikolova, Aleksandra Petrović, Radivoj Prodanović, Gorica Vuković, Jovanka Lević, Ilias Giannenas

**Affiliations:** 1 Department of Engineering Management in Biotechnology, Faculty of Economics and Engineering Management in Novi Sad, University Business Academy in Novi Sad, Cvećarska 2, 21000 Novi Sad, Serbia; rprodanovic@fimek.edu.rs; 2 Faculty of Veterinary Medicine, Agricultural University of Tirana, Kodor Kamez, 1000 Tirana, Albania; eklarisalika@yahoo.com; 3 Department of DETO, Section of Veterinary Science and Animal Production, University of Bari “Aldo Moro”, 70010 Valenzano, Italy; vincenzo.tufarelli@uniba.it; 4 Faculty of Agriculture, University of Novi Sad, Trg Dositeja Obradovića 8, 21000 Novi Sad, Serbia; petra@polj.uns.ac.rs; 5 Scientific Veterinary Institute Novi Sad, Rumenački put 20, 21000 Novi Sad, Serbia; 6 Institute of Animal Science, University “Ss. Cyril and Methodius”, Av. Ilinden 92/a, 1000 Skopje, North Macedonia; nikolova13@gmail.com; 7 Institute of Public Health of Belgrade, Bulevar despota Stefana 54a, 11000 Belgrade, Serbia; gorica.vukovic@zdravlje.org.rs; 8 Scientific Institute of Food Technology in Novi Sad, University of Novi Sad, Bulevar cara Lazara 1, 21000 Novi Sad, Serbia; jovanka.levic@fins.uns.ac.rs; 9 Laboratory of Nutrition, School of Veterinary Medicine, Faculty of Health Sciences, Aristotle University of Thessaloniki, 54124 Thessaloniki, Greece

**Keywords:** eggs, food, protein, health, antibiotics, residues, essential oils, tetracyclines, hens

## Abstract

The food of animal origin that is the most consumed is the table egg, but laying hens treated with antibiotics can produce eggs contaminated with antibiotic residues. Residues of antibiotics may present a risk for consumer health. Keeping in mind that laying hens almost always suffer from Mycoplasma (*Mycoplasma synoviae*), for which they are treated with antibiotics, high-quality egg production is even harder. Our research aimed to investigate the influence of three different antibiotics compared to the tea tree (*Melaleuca alternifolia*) essential oil administered to naturally infected laying hens with *M. synoviae*, on antibiotic residues in eggs as well as the egg nutritive and sensory qualities. A total of 20,000 laying hens, housed in one facility and divided into four lines each consisting of 5000 hens naturally infected with *M. synoviae*, was used. For the antimicrobial therapy, tetracycline (TC), oxytetracycline (OTC) and chlortetracycline (CTC) were used, respectively. As a control, tea tree essential oil (TT) was used. Based on the gained results all tetracyclines treatment residue values were significantly (*p* < 0.05) higher compared to the control treatment (TT), but without any significant differences (*p* > 0.05) between themselves. The results showed no differences in the nutritive and the sensory qualities of eggs between the control and the experimental treatments (*p* > 0.05). Keeping in mind the obtained results from this study, it can be concluded that tea tree essential oil could be successfully used as a natural antibiotic in the treatment of *M. synoviae*, without any adverse effects on table egg quality.

## 1. Introduction

The egg is the biggest cell, capable of producing life, with all the essential nutrients that could not be compared to any other food [1]. Eggs have relatively low energy and small concentrations of saturated fats (SFA) when compared to other products of animal origin [2,3]. It has been known that the eggs possess high-quality proteins [4] which could be also found in some dairy foods [2]. Over the years, a barrier to egg consumption was attributed to a belief that eggs are the main source of cholesterol, which is converted directly into serum cholesterol and thus increases the risk of cardiovascular diseases [5]. Egg nutritive and energetic values are much higher when compared to most other foods [6], including poultry meat [7]. A large boiled egg contains about 70 kcal energy, 5 g fat, of which 3.5 g is unsaturated (UFA) and 1.5 g is SFA [8]. Compared to sausages, hot dogs and other meat products this represents roughly 10% of the SFA content of the mentioned food animal products [9]. When it comes to egg protein quality and amino acid composition, they could be compared to beef meat [10], but with a significant difference because eggs have much higher bioavailability and better protein digestibility [11]. For a long time, it was believed that proteins were responsible for increased satiety [12]. Nowadays, this theory is proven by the results which demonstrate that increased protein concentration in eggs is responsible for satiety [13,14]. The same trend has been noticed in the promotion of weight loss programs for overweight or obese people regarding increasing satiety feeling with reduced energy intake [15]. Eggs have been shown to have high values of vitamins D and K, as well as increased concentrations of Se [3,16]. Researchers have indicated that this may be a reflection of the increased absorption of vitamins and minerals by hens in response to a change in feeding practices, with the replacement of feedstuffs of animal origin with vegetable oils [17] and other enhancements such as probiotics [18], prebiotics [19], enzymes [20], natural antioxidants [21], different botanicals [22,23,24,25,26,27,28] and the latest essential oils [24,29,30,31]. Finally, eggs are well renowned for being the principal source of good and highly desirable dietary cholesterol, next to the cholesterol and certain fatty acids from fish [32,33]. Keeping in mind that eggs are the most consumed high-quality food of animal origin, we must not forget that laying hens treated with antibiotics [34] can produce eggs contaminated with antibiotic residues. Residues of antibiotics may present a risk for consumer health. Laying hens almost always suffer from Mycoplasma and are consequently treated with antibiotics, which makes high-quality egg production even harder.

Today, the worldwide drug production is constantly rising and increasing. Almost half of the produced antibiotics are applied in food animal therapy and among them, tetracyclines have been the most applicable [35]. Because of their wide range of activities against Gram-positive and Gram-negative bacteria, rickettsia and viruses, as well as their relatively low toxicity, good tolerability and applications in various pharmaceutical forms, tetracycline antibiotics are widely used in veterinary medicine [36]. Tetracyclines are the secondary metabolism products of *Streptomyces* bacteria, though there are also semisynthetic tetracyclines in use [37]. Different researchers have shown that tetracyclines are effective against *Mycoplasma* [38], *Chlamydophila* [39] and *Rickettsia* spp. [40]. The most common and practical methods of tetracycline administration for poultry is through feed or water. The determination of antibiotic residues, including tetracyclines in egg samples, has become necessary to protect consumers and prevent the spread of antibiotic resistance [41]. In the case of positive results for tetracycline residues, above the allowed maximum levels (MRLs), confirmatory methods are applied, usually LC-MS/MS [42].

Plant extracts and spices as single compounds or as mixed preparations can have a significant role in supporting the performance, food quality and the health status of laying hens [23,28,30]. Although *Mycoplasma gallisepticum* has been largely freed from laying hens, it continues to persist in several generations of table egg-producing hens worldwide, resulting in losses in egg production, increased feed conversion rates and high mortality [43]. Over the years, adequate attention has not been paid to *M. synoviae*, until high economic losses in *M. synoviae* positive flocks were recorded [44]. This was the main reason for the increased interest in understanding the consequences of *M. synoviae* infections in laying hens. *M. synoviae* is an important pathogen of domestic poultry, causing economic losses to the table egg producers and is considered the second most important avian *Mycoplasma* sp. for domestic poultry [45]. *M. synoviae* is an egg transmitted pathogen that spreads horizontally through the respiratory pathways, usually affecting 100% of the hens in the facility [43,45]. Once infected, the hens become persistently infected with *M. synoviae* and remain carriers for life. This is the main reason for the antibiotic treatments [46], so the natural alternative in the form of essential oils has been more than necessary [47]. Most frequently, the infection occurs as a subclinical upper respiratory infection, which can progress to the respiratory lesions and become aggravated by other respiratory pathogens. Since 2000, problems in the table egg production have been more and more globally observed and are again caused by *M. synoviae* strains. The upper portion of the eggshell is translucent, thinner and more fragile to break, although sometimes the poor eggshell is defined genetically [48].

Some useful and important effects of the phytoadditives in animal nutrition include the stimulation of appetite and feed consumption, increased digestive enzyme secretions, immune response activations and antioxidant actions [21,26,28,29,31], as well as the potential use in parasite treatments, such as *Dermanyssus gallinae* [49,50]. The tea tree (*Melaleuca alternifolia*) is an aromatic and medicinal plant, found in many parts of the world which is significantly used in agriculture and pharmacy [30]. Every form of this plant, dry or fresh, has been used to replace chemical drugs in animal nutrition and veterinary therapy [22,24,51]. Prepared pulps or teas are used in traditional medicine to cure various diseases and relieve pain [34]. Numerous studies have been conducted to identify and quantify tea tree-oxygenated monoterpene and monoterpene hydrocarbons. The tea tree essential oil is rich in terpinen-4-ol, gamma-terpinene and alpha-terpinene [30,52]. Many studies reported the beneficial effects of essential oils and their chemical constituents, such as thymol and carvacrol, and their multiple biological activities: antioxidant, antimicrobial, antiviral, diaphoretic, expectorant, insecticidal and genotoxic [53]. Keeping in mind the characteristic aroma and chemical composition, *M. alternifolia* has been intensively used in agriculture as well as pharmaceutical and veterinary medicine, like many other plant species and products derived from their bioactive compounds [54]. Their antioxidant properties have proved to be effective in slowing lipid peroxidation, making them suitable in the production of table eggs and increasing the egg shelf life [21]. The composition of the tea tree bioactive compound extracts has been studied, in addition to their beneficial properties in in vivo experiments in laying hens’ nutrition for egg quality parameters, as well as their application in organic agriculture [51]. Since bioactive compounds represent secondary metabolites in plants, their concentration is influenced by many factors, genetic and environmental, so the continuous investigation and determination of their concentrations in plants are of high importance [55].

Therefore, based on the aforementioned information, our research aimed to investigate the influence of three different antibiotics compared to the tea tree (*M. alternifolia*) essential oil, in naturally infected laying hens with *M. synoviae* on the antibiotic residues in eggs and the eggs’ nutritive and sensory qualities.

## 2. Materials and Methods

Ethical Approval: The biological experiment with laying hens was performed following the EU legislation and principle of the Three Rs within Directive 2010/63/EU.

### 2.1. Experimental Design with Laying Hens

The experiment with laying hens was conducted under the principles of the European Union Strategy for the Protection and Welfare of Animals. A total of 20,000 Lohmann Brown hens aged 42 weeks were divided into four different treatment-diets supplemented with 100 mg/kg tea tree essential oil (TT) as a control treatment, followed by 100 mg/kg of each tetracycline (TC), oxytetracycline (OTC) and chlortetracycline (CTC), respectively (Table 1). The tea tree essential oil and antibiotics were applied in laying hens’ treatments trough the feed. Each treatment consisted of 5000 laying hens, respectively. The hens were housed in an environmentally controlled facility with a constant temperature of 22 °C. The environmental conditions in the facility were in line with hybrid specifications. All laying hens showed clinical signs of mycoplasmosis as nasal discharge and tracheal rales. In each treatment, 120 hens were marked and nasal swabs were collected aseptically. Marked hens were examined daily for 5 days after the application of the essential oil and antibiotics. The criterion for recovery was the absence of any nasal discharge when pressure was applied to the paranasal sinus.

Recovery percentage was calculated from the incidence of nasal discharge within the application days and after application. All the laying hens that had recovered were examined weekly for 4 weeks. Any hen that recovered but showed clinical signs in subsequent examination was classified as a relapse. The long-term cure rate for each treatment was calculated by subtracting the number of relapses to 33 days after treatment from the number of recovered hens recorded on day 5 after application according to the modified methodology previously described by Arzey and Arzey [56].

### 2.2. Tea Tree Essential Oil Gas Chromatography (GC) and GC–Mass Spectrometric (MS) Analysis

Gas chromatography (GC) and gas chromatography–mass spectrometric (GC–MS) analyses of the applied tea tree essential oil was performed using an Agilent 7890A GC equipped with an inert 5975C XL EI/CI mass spectrometer detector (MSD) and flame ionization detector (FID) connected by a capillary flow technology 2-way splitter with make-up. An HP-5MS capillary column (30 m × 0.25 mm × 0.25 μm) was used. The GC oven temperature was programmed from 60 °C to 300 °C at a rate of 3 °C min^−1^ and held for 15 min. Helium was used as the carrier gas at 16.255 psi (constant pressure mode). An auto-injection system (Agilent 7683B Series Injector) was employed to inject 1μL of the sample. The sample was analyzed in the splitless mode. The injector temperature was 300 °C and the detector temperature 300 °C. MS data were acquired in the EI mode with scan range 30–550 *m*/*z*, a source temperature of 230 °C and a quadrupole temperature of 150 °C; the solvent delay was 3 min.

### 2.3. Egg Samples Preparation for LC-MS/MS Residual Antibiotics

A total of 200 egg samples, 50 eggs from each treatment, were randomly selected. From each treatment 10 eggs were minced, making 5 samples each and then stored in polypropylene bottles at −20 °C until analyzed. The aliquot of 2.0 g of each sample was weighed into a 50 mL polypropylene tube, with 8 mL of acetonitrile added and the sample was homogenized. The sample was centrifuged and the supernatant was decanted into a 15 mL polypropylene tube. The volume of the extract was brought up to 10 mL with distilled water and 0.3 g of dispersive C18 was added to it. The supernatant was shaken for 1 min and then centrifuged for 5 min. Afterward, a 1 mL aliquot was diluted with 1 mL of distilled water and the extract was passed through a PTFE membrane filter. The final extract corresponded to a 0.1 g/mL sample equivalent. LC-MS/MS was performed with a spiking level of 0.1 mg/kg and the test data were evaluated based on recovery percentage (%) and relative standard deviation (SD).

### 2.4. Sample Preparation and Egg Nutritive and Sensory Analyses

Ten egg samples per treatment were collected randomly on the 33rd day after the application of the antibiotics and essential oil, to perform egg chemical and sensory analyses. The experimental data used for the analysis were performed according to the design by Brlek et al. [57] to limit the sample size, which was statistically correct for the estimation of the coefficients in a second degree least-squares approximating polynomial.

The nutritive quality of the eggs was determined according to the ISO recommended standards for moisture, protein, fat and ash contents, previously described by Puvača et al. [7], as well as for the pH values of egg albumen and egg yolk. The moisture content of the selected tissues was determined after drying the samples at 105 °C for 24 h to constant weight. Crude protein concentration was determined by the Kjeldahl method and ash was determined after burning at 550 °C ± 25 °C. Crude fat in the eggs was analyzed using the Soxhlet apparatus (SigmaAldrich, Buchs SG, Switzerland) with ether as a solvent. The data presented are means of 10 measurements.

The sensory evaluation of the table eggs was performed according to the methodology previously described by Spasevski et al. [3], only slightly modified, so a panel of 5 assessors experienced in a sensory analysis of the table eggs. Homogeneity and acceptability were evaluated by the 5-point category scale with endpoints labeled from 1 to 5 (Table 2). All properties were visually evaluated under laboratory conditions. The eggs were presented on plastic plates marked with a special code among assessors.

### 2.5. Statistical Analyses

The data gained from the investigation were analyzed by one-way variance analysis (ANOVA) within statistical software Statistica 13. When ANOVA showed statistical significance, Duncan’s multiple range post hoc test was used. A significant difference was registered at *p* < 0.05.

## 3. Results and Discussion

**Figure 1:**
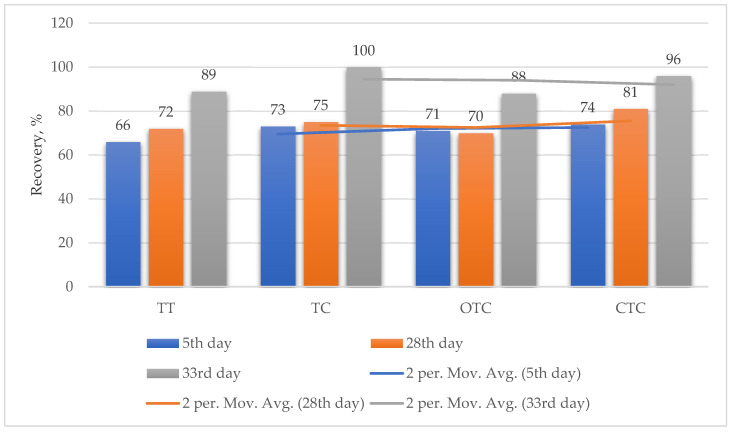
Effects of the application of the compound feed supplemented with 100 mg/kg tea tree essential oil (TT), tetracycline (TC), oxytetracycline (OTC) and chlortetracycline (CTC) on the laying hens’ recovery from mycoplasmosis.

The obtained results of the recovery treatments after the application of the tea tree essential oil and the antibiotics are shown in Figure 1, at periods of 1 to 33 days after the finished application of the essential oil and the antibiotics. All treatments showed a significantly high cure rate, without any statistically significant differences (*p* > 0.05) among the treatments. The cure rate of the laying hens was detected as soon as one day after the application of feed with 100 mg/kg tea tree essential oil (TT), tetracycline (TC), oxytetracycline (OTC) and chlortetracycline (CTC), which was maintained for 33 days, respectively. High long-term cure rates were achieved. The values of the calculated hens’ recovery and pooled standard error (SEp) varied from 66% to 89% (SEp 0.992) for TT, 73% to 100% (SEp 1.134) for TC, 71% to 88% (SEp 0.878) for OTC and 74% to 96% (SEp 0.937) for the CTC treatment, respectively (Figure 1).

According to Bradbury [58], laying hen mycoplasmosis may cause losses in egg production, while *M. synoviae* also affects broiler chickens and turkeys contributing toward respiratory disease, whereas in heavy layers it may cause synovitis and arthritis. Levisoh and Kleven [59] described *M. gallisepticum* as the most economically significant mycoplasma pathogen in poultry which possesses a worldwide distribution. The treatment efficacy with a single dose administration of five drugs at different dosages to the laying hens naturally infected with *M. gallisepticum* was prospected by Arzey and Arzey [56]. The antibiotics used in their research were tiamulin, tylosin, spiramycin, long-acting oxytetracycline and tylosin-dihydrostreptomycin. The cure was assessed by the absence of nasal discharge, as it was in our research. The results obtained in the research of Arzey and Arzey [56] were partly following some of our experimental treatments with antibiotics. According to the research of Catania et al. [60], *M. synoviae* is the most economically important pathogen in laying hens production. Their research has shown a significant difference in oviduct tropism between two *M. synoviae* strains in laying hens and a possible relationship to the production of eggshell apex abnormality in experimental conditions. In our research, eggshell abnormalities were recorded but without significant differences between the treatments. Researches related to the investigation of essential oils or different botanicals on *M. synoviae* in laying hens were performed in a very small number. Rehman et al. [61], have evaluated the efficacy of compound Chinese medicinal herbs against *M. synoviae* using different lab tests in mice and chickens. In their study, infected chickens supplemented with Chinese medicinal herbs in concentrations of 1; 1.5 and 2 mL/bird/day, for three days, showed that the concentration of 2 mL/bird/day had a better recovery response towards *M. synoviae*. The results of ELISA showed that *M. synoviae* were found in each treatment on day three. On days 15 and 18, no *M. synoviae* signs were observed. Based on these results, Rehman et al. [61] concluded that Chinese herbal medicine in the concentration of 2 mL/bird showed the best chicken recovery from *M. synoviae*. Our research showed that tea tree essential oil can be successfully applied as a replacement for antibiotic treatment very quickly, long term and with a high percentage (89%) of laying hens’ recovery from *M. synoviae*, which makes these findings very valuable. The reason for this was the presence of the phenolic compounds in the essential oil, which are a group of small molecules characterized by their structures having at least one phenol unit [62]. Based on their chemical structures, phenolic compounds can be divided into different subgroups, such as phenolic acids, flavonoids, tannins, coumarins, lignans, quinones, stilbenes and curcuminoids [63]. The results are presented in Table 3, which reveals the most dominant subgroup of the phenolic compound of the investigated essential oil. The conducted analyses showed that *M. alternifolia* essential oil wass the richest in terpinen-4-ol. The results of our research emphasized that the tea tree essential oil was rich in α-pinene (18.38%), limonene (7.55%) and γ-terpinene (14.01%). Alpha-pinene is an oxygenated monoterpene and a component of many aromatic dietary and medicinal plants [64]. Furthermore, α-pinene is a potent antioxidant that inhibits prostaglandin E1 and NF-κB and thereby contributes to its anti-inflammatory and anti-carcinogenic effects. Limonene is usually found in oils obtained from citrus plants, but it has also been detected as the most abundant terpene in cannabis, where it could be found in concentrations as high as 16% of the essential oil fraction [65]. Limonene is used for aromatic purposes in the cosmetic industry, as well as in pharmacology to facilitate the percutaneous drug transfer in vitro and in vivo [65]. Gamma-terpinene is a naturally occurring monoterpene hydrocarbon. It has been isolated from a variety of plant sources. This is a major component of various essential oils and has strong antioxidant activity [21], with the highest boiling point of the four known terpinine isomers (α-terpinine, β-terpinene, and δ-terpinine) [66]. It has a lemon-like or lime-like odor and is widely used in the food, flavor, pharmaceutical and perfume industries [66].

When used in the laying hens’ nutrition the essential oils enhanced the production of digestive secretions, stimulated blood circulation, exerted antioxidant properties, reduced levels of pathogenic bacteria and enhanced their immune status [23,30,68]. Ozek et al. [69] have shown the beneficial influences of the dietary herbal essential oil mixture and organic acid preparation on the laying traits, gastrointestinal tract characteristics, blood parameters and the immune response of laying hens in a hot summer season. With the wide range of available and affordable botanicals worldwide, the expanding horizons were revealed in the research field on essential oils and their bioactive principles in poultry nutrition [21,26,70].

Regarding the antibiotic residues, the results obtained in our study indicated significant differences (*p* < 0.05) between TT and the applied antibiotics in the experimental treatments (Table 4).

According to Commission Regulation (EU) No 37/2010 [71] of 22 December 2009 on pharmacologically active substances and their classification regarding MRLs in foodstuffs of animal origin, the maximum allowed residues of used antibiotics is 200 µg/kg (0.2 mg/kg). Our findings showed statistically significant differences (*p* < 0.05) in the residual antibiotics in table eggs. Nevertheless, the TT treatment served as a control treatment with the addition of the tea tree essential oil in the dietary amount of 100 mg/kg of hen weight. This treatment recorded 0.00 mg/kg of residual antibiotic concentration. The highest concentration of residual antibiotics in eggs (0.15 mg/kg) was recorded in the TC treatment with significant differences (*p* < 0.05) compared to the OTC treatment (0.08 mg/kg), but without significant differences (*p* > 0.05) when compared to the CTC treatment (0.11 mg/kg) treatment. Based on the aforementioned results of the laying hens’ recovery percentage, our results indicated that the tea tree essential oil could be usefully used in cure treatments for laying hens infected with *M. synoviae*, but without any adverse effects on table eggs. Moreover, we cannot neglect the positive effects of the used antibiotics with MRLs in eggs, which follow the Commission Regulation (EU) No 37/2010. These eggs can be safely used in human nutrition, keeping in mind the limits of antibiotics, but from the consumer point of view, these eggs are “not antibiotic-free”, which makes the tea tree essential oil much more desirable. Tea tree essential oil can be also used in green agriculture and organic agricultural production as a natural antibiotic and natural remedy for poultry illness and as a natural enhancer of production parameters [51].

Thirty years ago, Roudaut et al. [72] investigated various tetracyclines in different dosages in laying hens and the residual antibiotics in eggs. The research revealed that orally administered tetracycline through either drinking water (0.25 and 0.5 g/L for five days) or feed (300 and 600 ppm for seven days), and chlortetracycline through feed (600 ppm) induced the residue occurrences in eggs. The obtained results have exposed that antibiotic extractions from eggs were three-fold higher for tetracycline than for chlortetracycline. Roudaut et al. [72] confirmed that the elimination period from eggs lasted between six and 11 days for tetracycline and nine days for chlortetracycline after the antibiotic applications. Keeping in mind that antibiotics in products of animal origin and after excretion of these substances, in manure and in soil fertilized with that manure, may cause residues in food and the environment, the application of appropriate methodologies for the detection of oxytetracycline, tetracycline and chlortetracycline in eggs is of high importance. Sczesny et al. [73] have explained that the simple liquid extraction of the samples and HPLC separation, with collections of fractions on microtiter plates, could be analyzed by *Staphylococcus aureus* assay. When tetracycline residues in eggs were detected after the microbiological method, then with the same extraction procedure, LC−MS/MS could be used for the quantification of the residual antibiotics. Regarding the antibiotic’s negative effects on the human population, Jung et al. [74] conducted an in vitro study aimed to assess the impact of tetracycline on the human intestinal microbiome. According to their results, 0.15, 1.5, 15, and 150 μg/mL of tetracycline, after 24 h and 40 days of exposure of the fecal samples to tetracycline, in 3% human fecal suspensions, collected from three individuals, induced either no change or minor changes in the total bacterial 16S rRNA gene copies. This is important because the human intestinal microbiome, a generally stable microecosystem, could be potentially altered by the ingestion of antimicrobial drug residues in foods derived from animals. In our research, low residual concentrations of tetracycline in the table eggs from the treated hens could induce potential antimicrobial resistance in the human intestinal microbiome after consumption. Although doxycycline is a forbidden compound in laying hens, Gajda et al. [75] indicated that its residual concentration in eggs could depend on the type and time of the egg cooking procedures. Gajda et al. [75] showed that egg microwaving is the most effective manner in reducing doxycycline concentrations, over 50% after 4 min of heat treatment. Regarding the thermal processing of eggs, frying showed to be less effective compared to the microwaving (reduction <40% in 6 min), while boiling was the least effective (reduction <30% in 8 min) in doxycycline reduction, respectively. Therefore, the thermal treatments of eggs could not be the solutions for making table eggs safe for human consumption after the hens were treated with antibiotics.

**Figure 2:**
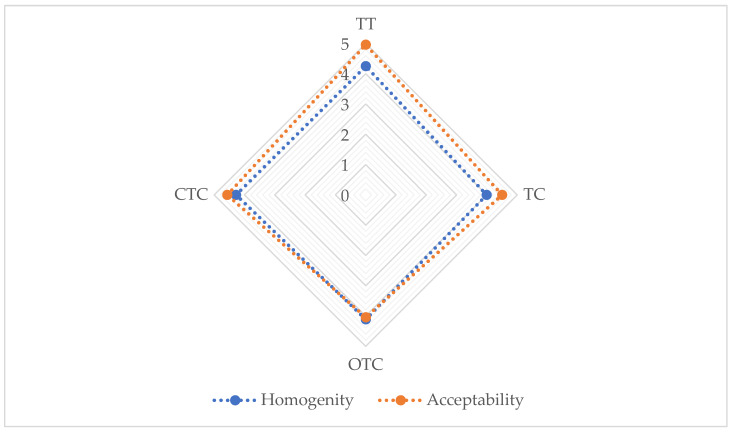
The sensory quality of the fresh table eggs of the laying hens. 0–5 Points described in Table 2; TT, CTC, TC, OTC Experimental treatments described in Table 1.

The results of the table eggs’ nutritional and sensory qualities are presented in Table 5 and Figure 2. From the results shown in Table 5, the treatments with the tea tree essential oil or antibiotics did not affect the nutritive quality of the eggs. The differences between the control and the experimental treatments were not statistically significant (*p* > 0.05). Moisture content ranged between 74.86% to 75.22%, total protein from 12.18% to 12.23%, total fat from 9.63% to 9.97% and crude ash from 0.87% to 0.91% (*p* > 0.05), respectively. The pH of the albumen and yolk were diverse and changed differently during storage [76]. The initial pH of the yolk was slightly acidic (5.9 to 6.2) and rose slightly during storage to about 6.8. The egg albumen pH was initially at the level of 7.6 and rose to 8.9 to 9.4 after storage due to CO_2_ losses through the shell [3]. The pH values for both albumen (7.22 to 7.68) and yolk (5.83 to 6.20) obtained in our experiment were following other studies [3,23,76], and were a confirmation of egg freshness.

Alagawany et al. [77] investigated the influence of probiotics as eco-friendly alternatives to antibiotics in poultry nutrition and concluded that they had positive effects on feed utilization, improved immunity and improved egg quality traits. Alternatively, Suresh et al. [78] provided a molecular perspective on the alternatives to antibiotics that have been proposed to date and their current trends, as well as the novel approaches, bearing in mind that the usage of antibiotic sub-therapeutic doses in laying hen treatments causes poultry pathogen antibiotic-resistant strains, such as *Salmonella*, *Campylobacter* and *Escherichia coli*. Gayathri et al. [79] investigated the effects of dietary supplementation with *Azadirachta indica* leaf meal compared to oxytetracycline on the egg nutrient profile and the production economics of laying hens. Their results led to the conclusion that the egg nutrient composition did not show any significant differences between the control and the antibiotic treatments, which was following the results obtained in our study. Furthermore, the same research [79] showed that up to 1% of *A. indica* in the diet of laying hens was beneficial in terms of the egg quality characteristics and production performance when compared to the antibiotic powder supplementation.

The results of the fresh eggs’ average homogeneity, as shown in Figure 2, ranged from 3.99 in the TC to 4.28 in the CTC treatment, without any statistically significant (*p* > 0.05) differences. This indicates that neither the addition of the tea tree essential oil nor the antibiotics had expressed their negative influence on the fresh table eggs’ homogeneity. The use of enrofloxacin residues in eggs and their influence on homogeneity and long-term stability were investigated [80]. The average values obtained in our study recorded a score of around 4, which indicated an “almost homogenous” description of the fresh table eggs, which presented an indicator of good sensory descriptive quality. When it comes to the scores of the fresh eggs’ acceptability, five trained panelists in our study scored the fresh eggs from 4.92 in the TT to 4.04 in the OTC treatment, without any statistically significant differences (*p* > 0.05). These results indicated the normal quality of the fresh eggs, with an average score higher than 4.5 (acceptable).

According to Pires et al. [81], essential oils preserve the internal egg quality in terms of pH values, which indicates that the application of essential oils could be an effective alternative for increasing the commercial eggs’ shelf life without adverse effects. Similar results have been shown in the review of Puvača et al. [30] regarding the beneficial effects of tea tree essential oil in poultry nutrition, as well in research regarding the influence of tea tree essential oil on the productive results of laying hens [51]. The influence of oregano essential oil and α-tocopheryl acetate on hen performance and egg quality was prospected by Giannenas et al. [23], who recorded significant differences (*p* < 0.05) only in yolk quality parameters [23].

## 4. Conclusions

Based on our findings, it could be concluded that the tea tree (*M. alternifolia*) essential oil, compared to different antibiotics, administered through the feed to naturally infected laying hens with *M. synoviae*, showed positive results. The used antimicrobial therapy with tetracycline, oxytetracycline and chlortetracycline demonstrated the presence of antibiotic residues in eggs, which were within the average range of the recommended maximum residue limits (MRLs). The eggs from the tea tree essential oil treatment were free from the antibiotics completely. The results emphasized no differences in the nutritive and sensory quality of the eggs between the control and the experimental treatments. Finally, and based on our results, the tea tree essential oil could be recommended as a successful natural antibiotic replacement in the treatment of *M. synoviae*, without any adverse effects on table egg quality. Nevertheless, although the antibiotic residues in the egg samples from this study were in the MRLs range, it should be highlighted that the excessive, inadequate and inappropriate usage of antibiotics for prophylactic purposes could have unwanted consequences for many years, due to their proven adverse influence on the environment and public health.

Certainly, this investigation showed positive results, but further research is necessary considering the lack of literature, actual research and in vivo investigations on this specific subject.

## Figures and Tables

**Table 1:** Experimental design with the laying hens.

Treatment		Concentration, mg/kg of Hen Weight	Average Daily Feed Consumption g/day	Average Hen Weight, kg
TT (control)	Tea tree essential oil	100.00	110.00	1.79 ± 0.12
TC	Tetracycline	100.00	110.00	1.78 ± 0.23
OTC	Oxytetracycline	100.00	110.00	1.79 ± 0.07
CTC	Chlortetracycline	100.00	110.00	1.81 ± 0.16

**Table 2:** The scoring method for the sensory quality of the fresh table eggs of the laying hens.

Homogeneity		Acceptability	
Points	Description	Points	Description
1	Highly non-homogenous	1	Unacceptable
2	Non-homogenous	2	Almost unacceptable
3	Slightly homogenous	3	Nor acceptable nor unacceptable
4	Almost homogenous	4	Almost acceptable
5	Homogenous	5	Acceptable

**Table 3:** Identified phenolic compounds of the tea tree essential oil, %.

Compound	RT ^2^	RI ^3^	*M. alternifolia* Composition ± SD
α-Thujene	5.636	922	1.10 ± 0.01
α-Pinene	5.862	930	18.38 ± 0.05
Camphene	6.241	945	0.08 ± 0.00
Sabinene	6.932	970	0.35 ± 0.00
β-Pinene	7.047	974	3.19 ± 0.01
Myrcene	7.428	988	0.45 ± 0.00
α-Phellandrene	7.900	1004	0.09 ± 0.00
Δ3-Carene	8.098	1009	0.09 ± 0.00
α-Terpinene	8.311	1015	2.35 ± 0.02
*p*-Cymene	8.598	1023	4.30 ± 0.00
Limonene	8.758	1027	7.55 ± 0.04
1,8-Cineole	8.835	1029	2.15 ± 0.01
β-(E)-Ocimene	9.439	1046	0.08 ± 0.00
γ-Terpinene	9.890	1058	14.01 ± 0.02
*p*-Mentha-2,4(8)-diene	10.891	1085	0.38 ± 0.00
Terpinolene	10.991	1088	3.56 ± 0.01
Borneol	14.240	1164	0.14 ± 0.00
Terpinen-4-ol	14.944	1180	38.53 ± 0.06
α-Terpineol	15.340	1190	2.16 ± 0.00
γ-Terpineol	15.606	1196	0.21 ± 0.00
(E)-Caryophyllene	25.448	1419	0.38 ± 0.00
NI ^1^			<0.50
Total peak area			564685150

^1^ Not identified; ^2^ Retention time; ^3^ Experimental retention indices based on n-alkane series under identical experimental conditions and comparison was done with the mass spectra library search NIST [67]; SD—standard deviation calculated for n (*n* = 3) gas chromatography–mass spectrometric (GC–MS) analysis.

**Table 4:** Effects of the applied tetracyclines on the recovery percentage and the residual antibiotics of the spiked samples in the eggs of the laying hens.

Treatment	Recovery	Spiked Level, mg/kg	Residual Antibiotic Concentrations, mg/kg
		0.1	
TT (control)	%	0.00 ^c^	0.00 ^c^
	SD	0.00	
TC	%	90.12 ^a^	0.15 ^a^
	SD	3.91	
OTC	%	85.25 ^b^	0.08 ^b^
	SD	5.12	
CTC	%	87.30 ^b^	0.11 ^a^
	SD	4.73	
*p* value		0.018	0.022
SEp		1.255	0.816

Different letters within a column indicate statistical differences (*p* < 0.05); SD—standard deviation; Sep—pooled standard error.

**Table 5:** Nutritional quality and the pH values of the whole fresh table eggs.

Treatment	Moisture, %	Protein, %	Fat, %	Ash, %	Egg pH Value	
					Albumen	Yolk
	Mean ± SD	Mean ± SD	Mean ± SD	Mean ± SD	Mean ± SD	Mean ± SD
TT (control)	75.22 ^a^ ± 0.62	12.23 ^a^ ± 0.18	9.92 ^a^ ± 0.46	0.91 ^a^ ± 0.02	7.50 ^a^ ± 0.12	5.91 ^a^ ± 0.86
TC	74.86 ^a^ ± 0.54	12.18 ^a^ ± 0.32	9.63 ^a^ ± 0.44	0.95 ^a^ ± 0.05	7.60 ^a^ ± 0.34	5.83 ^a^ ± 0.33
OTC	74.96 ^a^ ± 0.61	12.21 ^a^ ± 0.28	9.84 ^a^ ± 0.52	0.87 ^a^ ± 0.04	7.22 ^a^ ± 0.11	6.20 ^a^ ± 0.41
CTC	75.15 ^a^ ± 0.59	12.22 ^a^ ± 0.17	9.97 ^a^ ± 0.49	0.91 ^a^ ± 0.03	7.68 ^a^ ± 0.25	5.91 ^a^ ± 0.18
*p* value	0.833	0.624	0.891	0.929	0.522	0.609
SEp	1.408	1.665	1.209	1.765	1.384	0.997

Different letters within a column indicate statistical differences (*p* < 0.05); SD—standard deviation; SEp—pooled standard error.
